# Physiological Adaptations and Serum-Based Biomarker Dynamics During Multimodal Rehabilitation in Chronic Pain: Analysis of a Prospective Cohort Study

**DOI:** 10.3390/biom16060841

**Published:** 2026-06-09

**Authors:** Meike Meinzer, Markus Bassler, Franziska Kessemeier, Corinna Webering, Detlef Neumann, Heike Bähre, Ralf Lichtinghagen, Mathias Rhein, Johannes Achenbach, Christoph Gutenbrunner, Matthias Karst

**Affiliations:** 1 Department of Pharmacology, Hannover Medical School, 30625 Hannover, Germany; meike.meinzer@stud.mh-hannover.de (M.M.); neumann.detlef@mh-hannover.de (D.N.); 2 Institute for Social Medicine, Rehabilitation Sciencesand Health Services Research (ISRV), Nordhausen University of Applied Sciences, 99734 Nordhausen, Germany; msbassler@web.de; 3 Institute for Quality Assurance in Prevention and Rehabilitation (IQPR), German Sport University Cologne, 50933 Cologne, Germany; kessemeier@iqpr.de; 4 Rehabilitation Center Bad Pyrmont, 31812 Bad Pyrmont, Germany; corinna.webering@rehazentrum-bp.de; 5 Research Core Unit Metabolomics, Hannover Medical School, 30625 Hannover, Germany; baehre.heike@mh-hannover.de; 6 Institute of Clinical Chemistry and Central Laboratory of Hannover Medical School, 30625 Hannover, Germany; lichtinghagen.ralf@mh-hannover.de; 7 Laboratory for Molecular Neuroscience, Department of Psychiatry, Social Psychiatry and Psychotherapy, Hannover Medical School, 30625 Hannover, Germany; rhein.mathias@mh-hannover.de; 8 Department of Anaesthesia and Intensive Care Medicine, Royal Devon University Healthcare NHS-Foundation Trust, Barnstaple EX31 4JB, UK; johannes.achenbach@posteo.de; 9 Department of Rehabilitation and Sports Medicine, Hannover Medical School, 30625 Hannover, Germany; gutenbrunner.christoph@mh-hannover.de; 10 Department of Anesthesiology and Intensive Care Medicine, Pain Clinic, Hannover Medical School, 30625 Hannover, Germany; karst.matthias@mh-hannover.de

**Keywords:** chronic pain, multimodal pain rehabilitation, endocannabinoid system, biomarkers, 2-arachidonoylglycerol, treatment response

## Abstract

Background: Chronic pain is a multifactorial condition for which interdisciplinary multimodal rehabilitation is guideline-recommended, yet the biological mechanisms underlying treatment response remain incompletely understood and validated predictive biomarkers have not been established. Objective: This exploratory prospective cohort study examined clinical outcomes and circulating biomarker changes, encompassing the endocannabinoid system (ECS), inflammatory mediators, stress-regulatory markers, and metabolic parameters, in 410 patients with chronic pain of predominantly musculoskeletal etiology undergoing a standardized five-week rehabilitation program. Materials and Methods: Pain intensity and affective pain were assessed at baseline and end of rehabilitation; global performance of treatment (GPT) was additionally recorded. Serum analyses included anandamide (AEA), 2-arachidonoylglycerol (2-AG), IL-6, cortisol, IGF-1, BDNF, and leptin. Biomarker-outcome associations were examined via multiple regression analyses adjusted for demographics and biological and clinical confounders. Results: Statistically significant reductions were observed in pain intensity (−0.785 points, NRS; *p* < 0.001) and affective pain (−0.750 points; *p* < 0.001). IL-6 was associated with pain outcomes across time points. Higher baseline 2-AG independently predicted lower end-of-rehabilitation pain intensity, affective pain, and more favorable GPT. Greater AEA reductions were associated with favorable GPT. Conclusions: Baseline 2-AG emerges as a candidate predictor of treatment response, with lower pre-treatment levels potentially reflecting reduced stress-adaptive capacity, supporting inclusion of ECS markers in future controlled biomarker studies.

## 1. Introduction

Chronic pain, defined as pain persisting for more than three months, is a leading cause of disability worldwide [1]. It can be classified as nociceptive, neuropathic, inflammatory, or nociplastic [2]. The International Classification of Diseases (ICD) 11th revision further distinguishes chronic primary pain as a distinct disease entity from chronic secondary pain as a symptom of other conditions [2,3]. Chronic pain develops through interacting biological, psychological, and social factors, consistent with the biopsychosocial model [2,3].

Treatment options include pharmacological agents (non-steroidal anti-inflammatory drugs (NSAIDs), anticonvulsants, antidepressants, opioids), restorative therapies, psychological approaches, and complementary treatments such as acupuncture [4]. Despite this variety, many patients experience insufficient relief, and clinical practice often relies on trial-and-error rather than mechanism-based stratification [4]. Nevertheless, multimodal strategies are considered the treatment of choice in rehabilitation programs for chronic musculoskeletal pain [5].

Serum-based biomarkers could offer a path toward precision pain medicine by characterizing underlying mechanisms of chronic pain and predicting treatment response. A large-scale study involving a multidata set of participants with chronic pain recently found that biological and psychosocial markers predicted the onset and course of chronic pain mainly synergistically, while the predictive value of biomarkers for self-reported pain was low [6]. This gap might be filled by monitoring the activity of the endocannabinoid system (ECS), as it plays a multifaceted role in regulating pain, stress, and neuroimmune processes that modulate allostatic load [7]. Its main constituents are the cannabinoid 1 (CB1) and cannabinoid 2 (CB2) receptors, the endocannabinoids anandamide (arachidonoylethanolamine; AEA) and 2-arachidonoylglycerol (2-AG), and enzymes such as fatty acid amino hydrolase (FAAH) and monoacylglycerol lipase (MAGL) [8]. Altered ECS activity has been described in several chronic pain conditions, but its dynamics during rehabilitation remain underexplored [9]. Other circulating markers also reflect key processes in pain pathophysiology. Interleukin-6 (IL-6) promotes sensitization in peripheral and central pathways [10,11]. Cortisol indicates hypothalamic–pituitary–adrenal axis activity but findings in chronic pain are heterogeneous [12]. Insulin-like growth factor 1 (IGF-1) supports neuroplasticity with context-dependent pro-nociceptive or protective roles [13]. Brain-derived neurotrophic factor (BDNF) influences synaptic plasticity and has been linked to both sensitization and recovery [14]. Leptin bridges metabolic and inflammatory pathways and may link obesity with pain sensitization [15,16].

This exploratory study was designed to examine clinical changes during an interdisciplinary rehabilitation program for chronic musculoskeletal pain and to describe concurrent adaptations in endocannabinoid, inflammatory, metabolic, and neuroplasticity-related biomarkers. Rather than testing predefined causal mechanisms, the objective was to identify potential patterns of treatment response. This included the characterization of biomarker trajectories over time, changes in biomarker tonus, and associations between baseline biological profiles and variability in pain and functional outcomes. The analyses were intended to generate hypotheses for future stratification approaches and to contribute to a more detailed understanding of biomarker dynamics during rehabilitation.

## 2. Materials and Methods

An analysis of prospective data from a cohort study at the Reha-Zentrum Bad Pyrmont (Germany), a rehabilitation center for orthopedics, sports medicine, psychosomatics, and interdisciplinary pain therapy in Germany, was implemented as detailed below. The study was reported in accordance with the Strengthening the Reporting of Observational Studies in Epidemiology (STROBE) guidelines (Supplementary Material S1). The study was registered in the German Clinical Trials Register (DRKS00034556; date of registration 11 February 2025) and approved by the Ethics Committee of the Medical Association of Lower Saxony (ethical approval code Bo/32/2019, 26 June 2019). All patients provided informed consent and were included in pseudonymized data collection between April 2021 and January 2023.

### 2.1. Rehabilitation and Cohort

**Figure 1:**
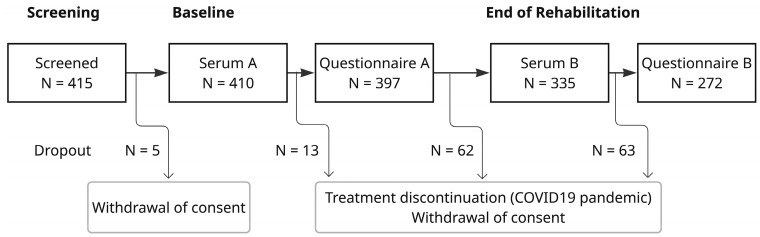
Flow diagram of recruitment and drop out.

Given the exploratory nature of the study, no formal sample size calculation was conducted and analyses were not designed for confirmatory hypothesis testing. To obtain a representative exploratory cohort, all patients admitted to the rehabilitation program during the study period were considered eligible and screened for inclusion. A total of 415 patients were screened, 410 provided informed consent, whereas 272 patients completed the last assessment (Figure 1). The high drop-out rate observed in this cohort was mainly due to treatment discontinuation because of the COVID-19 pandemic. Mean treatment duration was 34.5 days (SD = 4.6).

Patients were eligible for the interdisciplinary pain rehabilitation program at the psychosomatic–orthopedic rehabilitation center in Bad Pyrmont (Germany) if they had a chronic pain syndrome, needed rehabilitation, were capable of undergoing rehabilitation, and had received a commitment to cover costs from their pension insurance or health insurance provider (for more details see Supplementary Materials S2). Patient demographics and primary diagnoses are summarized in Table 1. Most patients had musculoskeletal disorders (n = 354), with a smaller subgroup with predominantly psychological diagnoses (n = 49), alongside comorbid mental health, cardiovascular, and metabolic disorders. The most common diagnoses classified according to ICD-10 were, M54.80 back pain (n = 52), M54.5 low back pain (n = 32), M15-M19 osteoarthritis (n = 28), and M51.1 lumbar disc disorder with radiculopathy (n = 22).

The 5-week rehabilitation program aimed to reduce symptom burden and support the restoration of work capacity through a structured multimodal approach. The manualized program comprised six group sessions focusing on pain-related psychoeducation and cognitive behavioral strategies; 12 interactive “athletic” group activities targeting muscular, coordinative, and cardiovascular resilience; and additional modules in relaxation training, ergotherapy, creative therapy, and music therapy.

### 2.2. Data Collection and Study Timeline

Pain intensity and physical function were assessed at baseline and end of rehabilitation (EOR). Fasted morning venous blood samples were collected; serum was separated for subsequent analyses. Clinical assessments at baseline and EOR employed the German Pain Questionnaire (GPQ) of the German Pain Society [17], a structured nine-page instrument capturing demographic data, pain characteristics, treatment history, comorbidities, and validated psychological and physical scales. The present analysis examined pain intensity, affective pain ratings and global performance of treatment as primary clinical outcomes, along with relevant demographic variables, and investigated associations between these outcomes and observed biomarker profiles.

Pain intensity was calculated as the mean of momentary, average, and maximum NRS ratings (0–10) and linear upscaled to 0–100 for analysis. Affective pain was derived by summing four descriptors (0–12), with scores ≥ 8 indicating elevated affective pain. At EOR a 5-point Likert scale was used to capture the global performance of treatment (GPT; 1 = excellent, 2 = very good, 3 = good, 4 = fair, 5 = poor), drawing on anchor-based approaches as established in pain research [18].

### 2.3. Biomarkers

The biomarker panel was selected to capture key biological systems relevant to pain regulation and adaptation to rehabilitation while maintaining a limited number of predictors to avoid model overparameterization in this exploratory cohort. Endocannabinoids (AEA, 2-AG) reflect activity of the endocannabinoid system involved in nociceptive modulation and stress regulation [7]; cortisol reflects HPA activity [12]; IL-6 indicates systemic inflammatory processes [11,19]; IGF-1 and BDNF reflect neuroplastic and regenerative mechanisms [13,14]; and leptin was included as a marker of metabolic and neuroendocrine signaling [15,16]. Biomarkers in serum were analyzed in the central laboratory of the Hannover Medical School (MHH; Deutsche Akkreditierungsstelle (German Accreditation Body) (DAkkS)-accredited according to Deutsches Institut für Normung (German Institute for Standardization) (DIN), Europäische Norm (European Standardization) (EN) and International Organization for Standardization (ISO) 15189:2014.), or the metabolomics laboratory at MHH (Table 2).

### 2.4. Statistical Methods

Statistical analyses were performed in JASP (v0.19; JASP Team, 2024). All tests were two-sided (α = 0.05). Normality was assessed via histogram and Q-Q plot inspection, supported by formal normality tests; parametric (*t*-test) or non-parametric (Mann–Whitney U) methods were applied accordingly.

Missing post-intervention data were handled using last observation carried forward (LOCF), a conservative approach selected in response to high attrition. Given only two assessment time points, LOCF assumes no post-baseline change in non-completers. Pearson’s correlation coefficients were calculated for covariate selection.

Associations between biomarkers and clinical outcomes were examined using multiple linear regression. Model diagnostics included Q–Q plots (residual normality), partial residual plots (linearity), and multicollinearity assessment (tolerance < 0.20; VIF > 10).

All analyses were exploratory and hypothesis-generating. For multiple testing, control false discovery rate (FDR) [19] was assessed within hypotheses, except within multiple linear regression models, where covariates were included for adjustment and confounding control [20]. FDR adjusted and unadjusted *p* were reported where applicable [21].

## 3. Results

### 3.1. Rehabilitation Efficacy According to Pain Intensity

**Figure 2:**
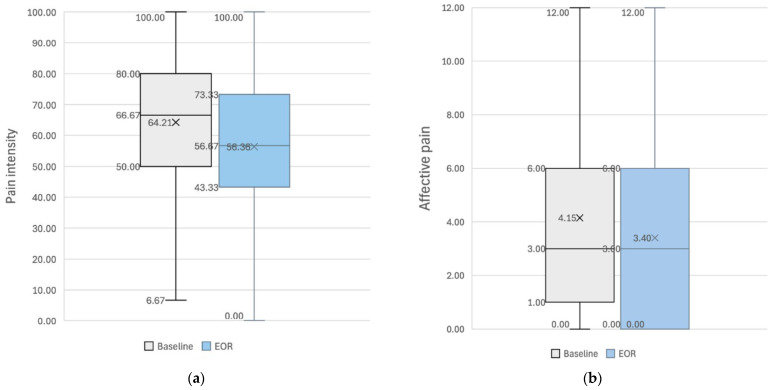
Box-and-whisker plot: Pain intensity (0–100) (**a**) and affective pain (0–12) (**b**); Box-and-whisker plot of paired samples baseline and EOR; interquartile range, median and mean; LOCF imputation applied.

Mean pain intensity decreased over the course of rehabilitation from 64.21 to 56.36 (t = 9.45, *p* < 0.00001, SE = 0.86; LOCF; FDR-P = 0.0001; Figure 2a). Affective pain ratings showed a similar reduction from a mean of 4.15 to 3.4 (t = 4.67, *p* = 0.000004, SE = 0.159; LOCF; FDR-P < 0.000004; Figure 2b).

### 3.2. BMI, Age, and Sex as Covariate Assessments for Biomarker Variability

At baseline, body mass index (BMI) showed correlations with most biomarkers except BDNF (2-AG: r = 0.172, *p* = 0.001; AEA: r = 0.344, *p* < 0.001; IL-6: r = 0.202, *p* < 0.001; cortisol: r = −0.119, *p* = 0.028; IGF-1: r = −0.148, *p* = 0.01; leptin: r = 0.52, *p* < 0.001; Supplementary Table S1). At EOR, BMI remained associated with 2-AG, AEA, and leptin (r = 0.312, 0.392, and 0.434; all *p* < 0.001).

Age was correlated at baseline with 2-AG (r = 0.141, *p* = 0.004), IL-6 (r = 0.129, *p* = 0.009), and IGF-1 (r = −0.295, *p* < 0.001). At EOR, associations with age were observed for 2-AG (r = 0.133, *p* = 0.015), cortisol (r = −0.147, *p* = 0.005), and IGF-1 (r = −0.36, *p* < 0.001) (Supplementary Table S2).

No statistically detectable differences by sex were observed (Supplementary Table S3). Given the observed associations, subsequent regression analyses included BMI and age as covariates to account for potential demographic influences on biomarker variability.

### 3.3. Baseline Biomarker Status and Clinical Status

Baseline IL-6 was the only one of the tested biomarkers or demographics associated with current pain intensity (B = 0.732, SE = 0.282, *p* = 0.01; FDR-P = 0.09, Supplementary Table S4). No baseline biomarker was associated with baseline affective pain (Supplementary Table S5).

### 3.4. Pre-Post Biomarker Changes

As presented in Table 3, AEA decreased from 0.93 to 0.77 nmol/L (*p* = 0.0001), cortisol from 19.04 to 18.24 µg/dL (*p* = 0.0016), and leptin from 3.85 to 3.33 ng/mL (*p* < 0.0001), while IGF-1 increased from 130.45 to 138.39 ng/mL (*p* = 0.032). No significant changes over time were observed in the serum concentrations of 2-AG, IL-6 and BDNF. Median 2-AG concentrations were below the reference interval reported for healthy populations. Median cortisol concentrations were above the reference interval at baseline but decreased over the course of rehabilitation towards reference values (Table 3). Similarly, BDNF concentrations were above the reference interval at baseline and showed a reduction towards reference values during rehabilitation.

### 3.5. Biomarker Intercorrelation

**Figure 3:**
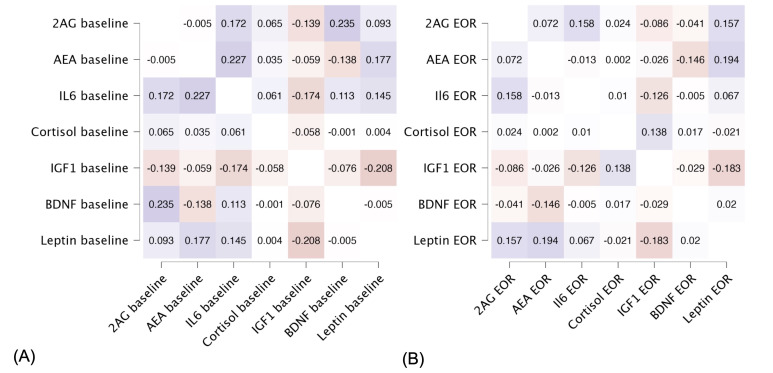
HeatMap of Pearson’s biomarker intercorrelations (**A**) Baseline and (**B**) EOR; r values depicted. Purple colors represent positive correlations, red colors represent negative correlations, and color saturation indicates the magnitude of the correlation coefficient (|r|).

At baseline, multiple significant correlations were observed (Supplementary Table S6, see Figure 3): leptin with IGF-1 (r = −0.208, *p* < 0.001), AEA (r = 0.177, *p* = 0.001), and IL-6 (r = 0.145, *p* = 0.007); BDNF with 2-AG (r = 0.235, *p* < 0.001), AEA (r = −0.138, *p* = 0.01), and IL-6 (r = 0.113, *p* = 0.034); IGF-1 with IL-6 (r = −0.147, *p* = 0.002), and 2-AG (r = −0.139, *p* = 0.012); and IL-6 with AEA (r = 0.227, *p* < 0.001), and 2-AG (r = 0.172, *p* < 0.001). Cortisol was not intercorrelated. AEA and 2-AG were not significantly intercorrelated. At EOR, intercorrelations decreased (Supplementary Table S7): leptin remained associated with AEA (r = 0.194, *p* < 0.001) and IGF-1 (r = −0.183, *p* < 0.001) and correlated with 2-AG (r = 0.157, *p* = 0.005); BDNF with AEA (r = −0.146, *p* = 0.011); IGF-1 with cortisol (r = 0.138, *p* = 0.009) and IL-6 (r = −0.126, *p* = 0.016); and IL-6 with 2-AG (r = 0.158, *p* = 0.004).

### 3.6. Biomarker Changes and Efficacy on Pain Intensity Ratings and Global Performance of Treatment (GPT)

Greater AEA (B = −0.734, SE = 0.312, *p* = 0.02, Supplementary Table S8) and leptin (B = −0.162, SE = 0.071, *p* = 0.024, Supplementary Table S8) reduction predicted more favorable GPT. No biomarker change was significantly associated with pain intensity or affective pain rating (Supplementary Tables S9 and S10).

### 3.7. Biomarker Tonus and Clinical Outcomes

#### 3.7.1. Baseline Biomarker Tone and GPT Efficacy

Higher baseline 2-AG was associated with more favorable GPT ratings at EOR (B = −0.122, SE = 0.061, *p* = 0.048), as was leptin (B = −0.064, SE = 0.028, *p* = 0.025) in models adjusted for BMI, age, and EOR pain intensity. These effects were observed despite pain intensity inclusion as covariate, which at EOR was independently associated with more negative GPT ratings (B = 0.025, SE = 0.003, *p* < 0.001; Supplementary Table S11).

At EOR, higher 2-AG showed an unadjusted association with better GPT (B = −0.209, SE = 0.109, *p* = 0.038), which was attenuated after adjustment for BMI, age, and pain intensity (Supplementary Table S12).

#### 3.7.2. Baseline Biomarker and EOR Pain Intensity

Higher pain intensity at EOR was associated with lower baseline 2-AG (B = −3.461, SE = 1.387, *p* = 0.013) and higher baseline IL-6 levels (B = 0.872, SE = 0.403, *p* = 0.031; Supplementary Table S13). Affective pain at EOR was likewise negatively associated with baseline 2-AG (B = −0.548, SE = 0.216, *p* = 0.012) and positively with IL-6 (B = 0.127, SE = 0.055, *p* = 0.047). After adjustment for BMI and age, only the association with baseline 2-AG remained statistically significant (B = −0.564, SE = 0.222, *p* = 0.012; Supplementary Table S14).

#### 3.7.3. EOR Biomarker and EOR Pain Intensity

After adjustment for covariates, pain intensity at EOR was associated only with BDNF (B = −0.518, SE = 0.257, *p* = 0.046; Supplementary Table S15). No significant associations were observed between any biomarker and affective pain at EOR (Supplementary Table S16).

## 4. Discussion

### 4.1. Rehabilitation and Pain Outcomes

Consistent with previous reports on interdisciplinary multimodal rehabilitation efficacy [5], patients showed statistically significant reductions in both pain intensity and affective pain, suggesting improvements across sensory and emotional pain dimensions. The mean pain intensity NRS reduction of 0.785 (10-point NRS) falls within the lower range of the reported Minimal Clinically Important Difference (MCID; 0.7–0.9) [29]. Mean affective pain decreased by 0.75 points, numerically corresponding to the lower MCID boundary for global pain, though specific validation for the affective GPQ scale 0–12 remains limited and results showed larger variation among all patients.

The average GPT score was 2.7 on the 5-point Likert scale (1 to 5), corresponding on average to ratings between “good” and “very good.” As MCID reflects the smallest improvement perceived as beneficial by patients, this overall evaluation provides contextual information for interpreting the observed symptom changes.

### 4.2. Biomarker Changes over Rehabilitation Course

AEA, cortisol, and leptin decreased over the rehabilitation period, whereas IGF-1 increased. The cortisol reduction is compatible with previously reported decreases in stress-related biomarkers during multimodal rehabilitation [30] and reflects normalization towards reference intervals of healthy populations. AEA, reported as elevated in some chronic pain populations and discussed as a compensatory antinociceptive signal [31,32], also declined, consistent with findings from comparable cohorts [33]. The IGF-1 increase aligns with the literature linking this marker to neuroplasticity and physical activity [34]. The leptin decrease exceeded what weight-change alone would predict, a pattern discussed in relation to metabolic and inflammatory processes [35].

IL-6, BDNF, and 2-AG showed no statistically detectable changes. IL-6 stability over short rehabilitation intervals has been previously reported [36]. The absence of detectable BDNF change may partly reflect the predominantly female cohort composition, given reported sex-dependent variation in BDNF responses, including attenuated changes in women [37].

### 4.3. Biomarker Intercorrelation

Biomarker intercorrelations and associations with demographic variables were less pronounced over the rehabilitation period. This reduction result may reflect differential responsiveness of the measured biological systems to the intervention, with heterogeneity in the temporal dynamics of change across systems. However, alternative explanations, including regression to the mean and reduced statistical power due to sample attrition, cannot be excluded. The observed reorganization of intercorrelation structure could be characterized more formally in future studies using a network biomarker framework, in which changes in inter-system coupling are quantified as properties of the network rather than as individual pairwise associations.

### 4.4. Baseline Biomarker Tonus and Clinical Baseline Status

Higher baseline IL-6 was positively associated with pain intensity (B = 0.732), consistent with its established role in nociceptive processing and central sensitization [38,39] and with reports identifying IL-6 as a biomarker for persistent pain [39]. Mechanistically, chronic IL-6 activation may divert metabolic resources from muscle maintenance, contributing to weakness and fatigue [40].

### 4.5. Predictors of Favorable GPT Associated with Biomarker Changes

Biomarker changes were not associated with reductions in pain intensity. However, greater AEA decreases during rehabilitation were significantly associated with more favorable GPT ratings. In line with the hypothesis of a compensatory increase in AEA in chronic pain [31], the observed decrease in AEA in patients with more favorable GPT scores could reflect a treatment-induced normalization of ECS tone. In this sense, AEA could represent a potential biomarker associated with biopsychological aspects of response, particularly self-perceived recovery.

A decrease in leptin levels during rehabilitation showed a weaker additional association with positive GPT scores, which could be consistent with reduced responsiveness to rehabilitation in individuals with higher metabolic burden [41].

### 4.6. Baseline Biomarkers and Clinical Status at EOR

Although 2-AG levels did not change significantly during rehabilitation, higher baseline 2-AG was negatively associated with pain intensity and affective pain at EOR and positively associated with GPT ratings, with associations remaining significant after adjustment for age and BMI. Notably, cohort-level 2-AG was below healthy reference ranges [22], contrasting with reports of elevated 2-AG in chronic pain conditions [42] and findings linking high acute-phase 2-AG to chronicity risk [43].

The effect observed might be mediated by stress. In this cohort, higher baseline 2-AG appeared protective, consistent with its role in HPA-axis modulation, where higher levels attenuate HPA activity [44,45]. Sub-reference 2-AG levels support a hypothesis of ECS hypofunction with reduced adaptive capacity to stress or therapeutic intervention.

That baseline status, rather than short-term dynamics, was the primary predictor of EOR outcomes suggests 2-AG may function as a trait-like indicator of recovery potential, with low baseline levels representing a candidate biomarker for unfavorable treatment trajectories. Future studies should examine whether interventions that upregulate 2-AG, including MAGL inhibition [46], physical exercise [47], or exogenous cannabinoid ligands, can modify these trajectories in chronic pain populations. This pattern is consistent with the results of a large-scale randomized clinical trial involving more than 800 patients, in which patients with chronic back pain were administered an exogenous endocannabinoid ligand, tetrahydrocannabinol, resulting in significantly greater symptom alleviation than placebo [48]. Moreover, this study showed that the global treatment effect, as measured by the Patient Global Impression of Change (PGIC) scale, was more pronounced than the purely analgesic effect, which was measured using the NRS [48]. Higher baseline IL-6 was independently associated with greater pain intensity at EOR, consistent with its established role in persistent pain states [39].

### 4.7. EOR Biomarker Tonus and Clinical Status at EOR

Whereas baseline IL-6 was strongly associated with pain intensity, at EOR, only BDNF showed a negative association with pain intensity. Elevated BDNF has been linked to neuroplastic processes following rehabilitation, including synaptic plasticity, descending inhibitory pathways, and cortical pain processing; functional recovery has frequently been accompanied by increased BDNF in prior studies [49]. However, BDNF’s role in pain remains complex: elevated levels have also been reported in chronic pain and linked to central sensitization [50,51]. Given that chronic pain has been conceptualized as involving maladaptive plasticity with learning-related components [52], the observed BDNF pattern, alongside multidimensional clinical improvements at EOR, may be compatible with adaptive neuroplastic processes.

### 4.8. Limitations

Medication data were not systematically recorded by treating physicians in the clinical database, and therefore pharmacological confounding cannot be excluded. Furthermore, only the primary diagnosis leading to rehabilitation admission was recorded, while no detailed assessment or classification of pain type, e.g., neuropathic pain, was performed. Further limitations of this study include the exploratory, single-center design. Inclusion of all eligible patients supports ecological validity; however, the multiple testing inherent in an exploratory analysis increases the risk of Type I error. To address this, FDR adjustment was applied to control the false discovery rate across hypothesis-testing analyses. Within individual multiple linear regression models, no within-model correction was applied, as predictor *p*-values were interpreted in the context of the overall model fit rather than as independent hypotheses; covariates were included primarily for confounder control, and coefficients are treated as descriptive and hypothesis-generating. Omitting within-model correction was a deliberate methodological choice to limit Type II error inflation in this exploratory context. All findings should therefore pend replication in clinical, adequately powered confirmatory studies.

Assessments were conducted at two time-points only, limiting the interpretability of temporal dynamics. The study was affected by a substantial dropout rate. Missing post-intervention data were handled using LOCF, assuming no change over the intervention period for non-completers. This conservative approach may underestimate true intervention effects and limit the generalizability of findings. Psychosocial variables and subgroup analyses were excluded to prevent model overfitting and have been addressed in a separate analysis [53]. Comedication and treatment history were not systematically recorded, potentially introducing uncontrolled confounding for biomarker variability and treatment response.

## 5. Conclusions

The results support the association between interdisciplinary multimodal rehabilitation and improvements in pain and global outcomes, which is consistent with the current treatment recommendations for chronic musculoskeletal pain. Clinical improvements were paralleled by alterations in selected biomarkers, including decreases in AEA, cortisol, and leptin, and an increase in IGF-1. While causality cannot be inferred, these temporal patterns are compatible with previously described stress-related, metabolic, and neuroplastic adaptations.

A reduction in baseline biomarker intercorrelations over time was observed, potentially reflecting changes in systemic organization across stress- and metabolism-related pathways. Patterns observed within the ECS included decreases in AEA levels, associated with more favorable GPT scores, whereas higher baseline 2-AG concentrations were associated with reduced affective and intensity pain ratings on the EOR scale and improved GPT scores, potentially mediated through modulation of the stress response axis. These findings suggest that dysfunction of the ECS, manifested by reduced basal 2-AG levels and diminished dynamics of AEA levels, could serve as a prognostic indicator of suboptimal treatment outcomes in patients with chronic pain.

The ECS appears to contribute meaningfully to variability in rehabilitation trajectories, and the integration of ECS biomarkers into rehabilitation research may support differentiated patient characterization and a mechanistic foundation for stratified approaches in chronic pain management.

Future studies should incorporate repeated multidimensional assessments and an expanded biomarker panel, including enzymatic regulators, additional endocannabinoid ligands, and genetic polymorphisms affecting endocannabinoid signaling, to refine mechanistic understanding and support biomarker-based prediction of individual treatment responsiveness.

## Figures and Tables

**Table 1:** Description of baseline cohort patient characteristics.

	Total	Male	Female	
Enrolled (%)	N = 410	N = 159 (38.8)	N = 251 (61.2)	
Age (mean ± SD)	52 ± 8.4	52 ± 8.1	53 ± 8.5	
BMI (mean ± SD)	29.6 ± 8.3	29.6 ± 9.2	29.5 ± 7.7	
Primary diagnosis				
ICD	Category		N	%
M	Diseases of the musculoskeletal system and connective tissue		340	82.9
F	Mental and behavioral disorders		47	11.5
S	Injuries		7	1.7
G	Nervous System disorders		3	0.7
T	Injury/poisoning consequences		2	0.5
Missing	-		11	2.7

BMI = Body Mass Index.

**Table 2:** Methods of analysis of included biomarkers.

Biomarker	Methods of Analysis	Location of Analysis
Endocannabinoids 2-AG; AEA	The quantification of AEA and 2-AG was performed using LC-MS/MS. A QRTAP5500 (Sciex, Framingham, MA, USA) was used as the mass spectrometer, and the LC was a system from Shimadzu (Shimadzu, Duisburg, Germany).	Metabolomics laboratory of Hannover Medical School
Cortisol	Electrochemiluminescence Immunoassay (ECLIA), cobas 8000 (module cobas e 801, Roche Diagnostics GmbH, Sandhoferstr. 116, 68305 Mannheim, Germany. Packungsinformation Cobas Elecsys Cortisol II.); procedures per manufacturer specifications [Roche Cortisol] and LVZ MHH internal validation guidelines [MHH LVZ 2025]	Institute of Clinical Chemistry and Central Laboratory of Hannover Medical School
IL-6	Electrochemiluminescence Immunoassay (ECLIA), cobas 8000 (Roche Diagnostics GmbH. Packungsinformation Cobas Elecsys IL-6. Mannheim); procedures per manufacturer specifications [Roche IL6], and LVZ MHH internal validation guidelines [MHH LVZ 2025]	Institute of Clinical Chemistry and Central Laboratory of Hannover Medical School
IGF-1	Electrochemiluminescence Immunoassay (ECLIA), cobas 8000 (Roche Diagnostics GmbH. Packungsinformation Cobas Elecsys IGF-1. Mannheim); procedures per manufacturer specifications [Roche IGF1] and LVZ MHH internal validation guidelines [MHH LVZ 2025]	Institute of Clinical Chemistry and Central Laboratory of Hannover Medical School
BDNF	An enzyme immunoassay was performed using the Human BDNF ELISA kit [ Boster Biological Technology, 3942 Valley Ave, Pleasanton, CA 94566, USA Human BDNF ELISA Kit PicoKine^®^ (Cat. No. EK0307) USA]	Institute of Clinical Chemistry and Central Laboratory of Hannover Medical School
Leptin	An enzyme immunoassay was performed using the Human Leptin ELISA kit [RayBiotech Inc. RayBio^®^ Human Leptin ELISA Kit (Cat. No. ELH-Leptin) User Manual. Norcross, GA, USA.]	Institute of Clinical Chemistry and Central Laboratory of Hannover Medical School

AEA = Arachidonoylethanolamine, 2-AG = 2-arachidonoylglycerol, BDNF = Brain-derived neurotrophic factor, IGF-1 = Insulin-like growth factor 1, IL-6 = Interleukin-6; 37. Medizinische Hochschule Hannover. Leistungsverzeichnis (LVZ) Zentrallabor. Version 21.0.

**Table 3:** Pre–post comparison biomarker tonus, repetition of significant markers with LOCF imputation of missing data.

Biomarker		n	M (SD)	t	*p*	FDR-p	RI	Values vs. RI *
AEA	Pre	410	0.93 (0.33)	9.9078	0.0001	0.0004	0.5–1.5 [22]	⇔
	Post		0.77 (0.28)					
2-AG	Pre	410	1.78 (1.37)	0.1195	0.9	0.9	F 3.7–39.3 M 7.2–66.7 [22]	↓
	Post		1.63 (1.17)					
BMI	Pre	410	29.28 (5.95)	1.0585	0.2901	0.39	18.5–30 [23]	⇔
	Post		28.95 (5.94)					
IL-6	Pre	409	2.68 (3.95)	0.033348	0.9	0.9	F ≤ 15.96 M ≤ 14.61 [24]	⇔
	Post		2.73 (7.58)					
Cortisol	Pre	409	19.04 (4.8)	3.1855	0.0016	0.0043	6–18.4 (morning) [25]	↑
	Post		18.24 (4.77)					
IGF-1	Pre	324	130.45 (45.52)	2.1484	0.0321	0.39	500 (puberty)–100 (80Y) [26]	⇔
	Post		138.39 (47.79)					
BDNF	Pre	349	21.45 (8.37)	−1.79	0.074	0.12	1.5–18.5 [27]	↑
	Post		20.8 (8.47)					
Leptin	Pre	343	3.85 (2.94)	7.8848	<0.0001	0.0004	F: 3.60–54.86 M: 0.33–19.85 [28]	⇔

*t*-test for two dependent means; Mean values (M) are depicted in nmol/L (AEA, 2-AG), µg/dL (cortisol), ng/mL (BDNF, IGF-1, leptin), pg/mL (IL-6) and (kg/m2) (BMI); * relation to reference interval: ⇔ within the reference interval, ↑ above the reference interval, ↓ below the reference interval; abbreviations: AEA = Arachidonoylethanolamine, 2-AG = 2-arachidonoylglycerol, BMI = Body Mass Index, BDNF = Brain-derived neurotrophic factor, FDR = False Discovery Rate, IGF-1 = Insulin-like growth factor 1, IL-6 = Interleukin-6, LOCF = Last observation carried forward, RI = Reference Interval.

## Data Availability

The datasets used and/or analyzed during the current study are available from the corresponding author on reasonable request for data privacy reasons.

## Supplementary Materials

The following supporting information can be downloaded at: https://www.mdpi.com/article/10.3390/biom16060841/s1, Supplementary Materials are available. Supplementary Material S1: STROBE Checklist. Supplementary Material S2: Supplementary information about the rehabilitation program. Table S1. Pearson’s correlations, demographics and biomarkers: baseline. Table S2. Pearson’s correlations, demographics and biomarkers: EOR. Table S3. Group differences male and female sex for all biomarkers. Table S4. Multiple linear regression model baseline biomarkers and baseline pain intensity + BMI + age. Table S5. Multiple linear regression model baseline biomarkers and baseline affective pain + BMI + age. Table S6. Pearson’s correlations: baseline biomarkers. Table S7. Pearson’s correlations: EOR biomarkers. Table S8. Multiple linear regression model significant pre-post changes and GPT. Table S9. Multiple linear regression significant biomarker changes (pre-post) and GPQ pain intensity change pre-post. Table S10. Multiple linear regression significant biomarker changes (pre-post) and GPQ affective pain intensity change pre-post. Table S11. Multiple linear regression GPT and baseline biomarkers + BMI + EOR pain intensity. Table S12. Multiple linear regression GPT and EOR biomarkers + BMI + EOR pain intensity. Table S13. Multiple linear regression EOR GPQ pain intensity and all baseline biomarkers + BMI, Age. Table S14. Multiple linear regression GPQ EOR affective pain and all baseline biomarkers, BMI. Table S15. Multiple linear regression EOR GPS Pain Intensity and all EOR biomarkers + BMI, Age. Table S16. Multiple linear regression GPS EOR affective pain and all EOR biomarkers + BMI, Age.

## Abbreviations

The following abbreviations are used in this manuscript:

2-AG	2-arachidonoylglycerol
AEA	Arachidonoylethanolamine/Anandamide
BDNF	Brain-derived neurotrophic factor
BMI	Body mass index
CB1; CB2 receptor	Cannabinoid receptor 1, 2
CNS	Central nervous system
DAkkS	Deutsche Akkreditierungsstelle (German Accreditation Body)
DIN	Deutsches Institut für Normung (German Institute for Standardization)
DRKS	Deutsches Register Klinischer Studien (German Clinical Trials Register)
ECLIA	Electrochemiluminescence Immunoassay
ELISA	Enzyme-Linked Immunosorbent Assay
ECS	Endocannabinoid system
EOR	End of rehabilitation
EN	Europäische Normung (European Standadization)
FAAH	Fatty acid amino hydrolase
GPS	German Pain Society („Deutsche Schmerzgesellschaft e.V.“)
GPQ ("DSF")	German Pain Questionnaire („Deutscher Schmerzfragebogen“)
HPA	Hypothalamic–pituitary–adrenal
IGF-1	Insulin-like growth factor 1
IGF-1R	Insulin-like growth factor 1 receptor
ICD	International Classification of Diseases
ISO	International Organization for Standardization
DIN EN ISO 15189	International Standard for Medical Laboratories
IL-6	Interleukin-6
IL-6R	Interleukin-6-receptor
LOCF	Last Observation Carried Forward
MHH	Medizinische Hochschule Hannover (Hannover Medical School)
MAGL	Monoacylglycerol lipase
NSAIDs	Non-steroidal anti-inflammatory drugs
NRS	Numeric rating scale
GPT	Global Performance of Treatment
Q-Q plots	Quantile-Quantile plots
SD	Standard deviation
