# Supplementary material for: Physiological Adaptations and Serum-Based Biomarker Dynamics During Multimodal Rehabilitation in Chronic Pain: Analysis of a Prospective Cohort Study
Source: Biomolecules. 2026 Jun 9;16(6):841. doi: 10.3390/biom16060841 (PMC13297331; doi:10.3390/biom16060841)
Supplement: Supplementary file 1 [file biomolecules-16-00841-s001.zip › biomolecules-4346300-supplementary.pdf]

**S1 STROBE Checklist**

STROBE Statement—Checklist of items that should be included in reports of ***cohort studies***

| | Item No | Recommendation | Page No |
|---|---|---|---|
| **Title and abstract** | 1 | (*a*) Indicate the study's design with a commonly used term in the title or the abstract | 1 |
| | | (*b*) Provide in the abstract an informative and balanced summary of what was done and what was found | 1 |
| **Introduction** | | | |
| Background/rationale | 2 | Explain the scientific background and rationale for the investigation being reported | 2 |
| Objectives | 3 | State specific objectives, including any prespecified hypotheses | 2-3 |
| **Methods** | | | |
| Study design | 4 | Present key elements of study design early in the paper | 4 |
| Setting | 5 | Describe the setting, locations, and relevant dates, including periods of recruitment, exposure, follow-up, and data collection | 4-5 |
| Participants | 6 | (*a*) Give the eligibility criteria, and the sources and methods of selection of participants. Describe methods of follow-up | 4 |
| | | (*b*) For matched studies, give matching criteria and number of exposed and unexposed | - |
| Variables | 7 | Clearly define all outcomes, exposures, predictors, potential confounders, and effect modifiers. Give diagnostic criteria, if applicable | 5 |
| Data sources/ measurement | 8* | For each variable of interest, give sources of data and details of methods of assessment (measurement). Describe comparability of assessment methods if there is more than one group | 5-6 |
| Bias | 9 | Describe any efforts to address potential sources of bias | 6 |
| Study size | 10 | Explain how the study size was arrived at | 4 |
| Quantitative variables | 11 | Explain how quantitative variables were handled in the analyses. If applicable, describe which groupings were chosen and why | 5-6 |
| Statistical methods | 12 | (*a*) Describe all statistical methods, including those used to control for confounding | 6 |
| | | (*b*) Describe any methods used to examine subgroups and interactions | 6 |
| | | (*c*) Explain how missing data were addressed | 6 |
| | | (*d*) If applicable, explain how loss to follow-up was addressed | 6 |
| | | (*e*) Describe any sensitivity analyses | 6 |
| **Results** | | | |
| Participants | 13* | (a) Report numbers of individuals at each stage of study—eg numbers potentially eligible, examined for eligibility, confirmed eligible, included in the study, completing follow-up, and analysed | 4 |
| | | (b) Give reasons for non-participation at each stage | 4 |
| | | (c) Consider use of a flow diagram | 4 |
| Descriptive data | 14* | (a) Give characteristics of study participants (eg demographic, clinical, social) and information on exposures and potential confounders | 4-5 |
| | | (b) Indicate number of participants with missing data for each variable of interest | 4-5 |
| | | (c) Summarise follow-up time (eg, average and total amount) | 4 |
| Outcome data | 15* | Report numbers of outcome events or summary measures over time | 4 |

| | | | |
|---|---|---|---|
| Main results | 16 | (*a*) Give unadjusted estimates and, if applicable, confounder-adjusted estimates and their precision (eg, 95% confidence interval). Make clear which confounders were adjusted for and why they were included | 7-10 |
| | | (*b*) Report category boundaries when continuous variables were categorized | 1-10 |
| | | (*c*) If relevant, consider translating estimates of relative risk into absolute risk for a meaningful time period | - |

| | | | |
|---|---|---|---|
| Other analyses | 17 | Report other analyses done—eg analyses of subgroups and interactions, and sensitivity analyses | - |
| **Discussion** | | | |
| Key results | 18 | Summarise key results with reference to study objectives | 10-12 |
| Limitations | 19 | Discuss limitations of the study, taking into account sources of potential bias or imprecision. Discuss both direction and magnitude of any potential bias | 12-13 |
| Interpretation | 20 | Give a cautious overall interpretation of results considering objectives, limitations, multiplicity of analyses, results from similar studies, and other relevant evidence | 10-12 |
| Generalisability | 21 | Discuss the generalisability (external validity) of the study results | 12-13 |
| **Other information** | | | |
| Funding | 22 | Give the source of funding and the role of the funders for the present study and, if applicable, for the original study on which the present article is based | 14 |

*Give information separately for exposed and unexposed groups.

**Note:** An Explanation and Elaboration article discusses each checklist item and gives methodological background and published examples of transparent reporting. The STROBE checklist is best used in conjunction with this article (freely available on the Web sites of PLoS Medicine at http://www.plosmedicine.org/, Annals of Internal Medicine at http://www.annals.org/, and Epidemiology at http://www.epidem.com/). Information on the STROBE Initiative is available at http://www.strobe-statement.org.

**S2 Rehabilitation Concept**

Treatment approach of the rehabilitation program.

Participants were treated as part of an interdisciplinary, multimodal pain rehabilitation program at a German inpatient rehabilitation center funded by the German Pension Insurance. The program followed a biopsychosocial rehabilitation model for chronic pain and was based on the International Classification of Functioning, Disability, and Health (ICF), incorporating somatic, psychological, behavioral, and social aspects of pain-related disability. The treatment aimed not only at pain relief but also at improving functional ability, participation, self-management, and work capacity.

Prior to admission, medical records and referral documents were reviewed by an interdisciplinary team to assess the indication for treatment and the need for rehabilitation. Upon admission, all patients underwent a structured medical and psychological evaluation, which included a physical examination, a psychological assessment of pain, an assessment of participation and activity levels, and a work-related context assessment. Rehabilitation goals were established jointly with the patients according to the principles of shared decision-making and were regularly adjusted throughout the course of treatment.

The rehabilitation program was carried out by an interdisciplinary team consisting of specialists in pain medicine and rehabilitation, psychologists/psychotherapists, physical therapists, pain physical therapists, sports therapists, occupational therapists, nursing staff and social workers. Treatment planning and adjustments were coordinated during regular interdisciplinary case conferences, in which clinical findings, functional limitations, psychosocial factors, and rehabilitation progress were discussed together.

The program combined several coordinated treatment components, which were carried out simultaneously rather than sequentially. The core elements included medical pain management and medication review, individual and group psychotherapy focused on pain coping and behavioral change, physical therapy and pain-specific physical therapy, exercise and sports therapy, occupational therapy, patient education, and social counseling. Psychotherapeutic interventions aimed to strengthen *a* biopsychosocial understanding of pain, reduce maladaptive coping patterns and anxiety-avoidance behaviors, improve emotional regulation and stress management, and reinforce pain coping strategies (e.g., attention redirection, imagery, self-instructions, and behavioral activation). Physical therapy and movement-based interventions focused on restoring confidence in movement, increasing functional activity, improving body awareness, and promoting graded physical activation and self-management. Occupational therapy addressed functional abilities, activity limitations, self-care, participation, and occupational functioning. Social counseling addressed vocational participation, reintegration into the work process, and socio-medical issues.

Treatment was individually tailored to the patients' symptom burden, psychosocial complexity, and rehabilitation goals. Depending on whether somatic or psychosocial factors were relatively more predominant, the treatment focus was adjusted, while maintaining a multimodal, interdisciplinary approach. Rehabilitation lasted approximately 4–5 weeks, with treatment plans continuously adjusted based on interdisciplinary reviews and the patients' progress.

In addition, the detailed treatment protocol is available in German and can be requested from the corresponding author.

## Supplementary Tables

*Table S1. Pearson's correlations Demographics and biomarkers Baseline*

| Variable | | 2-AG baseline | AEA baseline | IL-6 baseline | Cortisol baseline | IGF-1 baseline | BDNF baseline | Leptin baseline |
|---|---|---|---|---|---|---|---|---|
| BMI baseline | Pearson's r | 0.172 | 0.344 | 0.202 | -0.119 | -0.148 | 0.002 | 0.520 |
| | p-value | 0.001* | < .001* | < .001* | 0.028° | 0.010* | 0.968 | < .001* |
| | FDR p | 0.005 | 0.005 | 0.005 | 0.07° | 0.03 | 0.9 | 0.005 |
| Age | Pearson's r | 0.141 | 0.049 | 0.129 | -0.095 | -0.295 | 0.043 | 0.029 |
| | p-value | 0.004* | 0.324 | 0.009* | 0.054 | < .001* | 0.429 | 0.592 |
| | FDR p | 0.017 | 0.5 | 0.03 | 0.13 | 0.005 | 0.65 | 0.75 |

*Indicates significances; FDR (false discovery rate) adjusted p following Benjamin-Hochberg (1995) potential false positives marked with (°); AEA= Arachidonoylethanolamine, 2-AG=2-arachidonoylglycerol, BMI=Body Mass Index, BDNF=Brain-derived neurotrophic factor, IGF-1= Insulin-like growth factor 1, IL-6= Interleukin-6

*Table S2. Pearson's correlations Demographics and biomarkers EOR*

| Variable | | 2-AG EOR | AEA EOR | IL-6 EOR | Cortisol EOR | IGF-1 EOR | BDNF EOR | Leptin EOR |
|---|---|---|---|---|---|---|---|---|
| BMI EOR | Pearson's r | 0.312 | 0.392 | 0.053 | -0.098 | -0.071 | -0.025 | 0.434 |
| | p-value | < .001* | < .001* | 0.420 | 0.132 | 0.277 | 0.706 | < .001* |
| | FDR p | 0.005 | 0.005 | 0.65 | 0.3 | 0.5 | 0.83 | 0.005 |
| Age | Pearson's r | 0.133 | 0.038 | 0.041 | -0.147 | -0.360 | -0.038 | 0.035 |
| | p-value | 0.015* | 0.484 | 0.437 | 0.005* | < .001* | 0.493 | 0.515 |
| | FDR p | 0.04 | 0.68 | 0.65 | 0.02 | 0.005 | 0.69 | 0.69 |

*Indicates significances; FDR (false discovery rate) adjusted p following Benjamin-Hochberg (1995) potential false positives marked with (°); AEA= Arachidonoylethanolamine, 2-AG=2-arachidonoylglycerol, BMI=Body Mass Index, BDNF=Brain-derived neurotrophic factor, EOR=End of rehabilitation, IGF-1= Insulin-like growth factor 1, IL-6= Interleukin-6

*Table S3. Group differences male and female sex for all biomarkers*

| | | **Male** | **Female** |
|---|---|---|---|
| AEA baseline (nmol/L) | N | N = 159 | N = 251 |
| | Median (SD) | 0.925 (0.325) | 0.940 (0.334) |
| | p | 0.849 | |
| | FDR p | 0.92 | |
| 2-AG baseline (nmol/l) | N | N = 159 | N = 251 |
| | Median (SD) | 1.860 (1.470) | 1.733 (1.300) |
| | p | 0.602 | |
| | FDR p | 0.75 | |
| IL-6 baseline (pg/ml) | N | 159 | 250 |
| | Median (SD) | 2.44 (3.14) | 2.82 (4.38) |
| | p | 0.326 | |
| | FDR p | 0.53 | |
| Cortisol baseline (µg/dL) | N | 159 | 250 |
| | Median (SD) | 19.48 (5.22) | 18.77 (4.51) |
| | p | 0.263 | |
| | FDR p | 0.5 | |
| IGF-1 baseline (ng/ml) | N | 128 | 196 |
| | Median (SD) | 132.41 (45.72) | 129.17 (45.46) |
| | p | 0.588 | |
| | FDR p | 0.75 | |
| BDNF baseline (ng/ml) | N | 137 | 212 |
| | Median (SD) | 20.91 (8.29) | 21.8 (8.41) |

| | | | |
|---|---|---|---|
| | p | 0.299 | |
| | FDR p | 0.5 | |
| Leptin baseline (ng/ml) | N | 133 | 210 |
| | Median (SD) | 3.615 (2.8) | 4.0 (3.02) |
| | p | 0.253 | |
| | FDR p | 0.49 | |
| AEA EOR (nmol/L) | N | N = 130 | N = 205 |
| | Median (SD) | 0.777 (0.285) | 0.765 (0.280) |
| | p | 0.73 | |
| | FDR p | 0.84 | |
| 2-AG EOR (nmol/L) | N | N = 130 | N = 205 |
| | Median (SD) | 1.603 (1.213) | 1.648 (1.148) |
| | p | 0.559 | |
| | FDR p | 0.74 | |
| IL-6 EOR (pg/ml) | N | 141 | 222 |
| | Median (SD) | 2.1 (2.35) | 3.14 (9.5) |
| | p | 0.255 | |
| | FDR p | 0.49 | |
| Cortisol EOR (µg/dl) | N | 141 | 222 |
| | Median (SD) | 18.32 (4.42) | 18.0 (4.99) |
| | p | 0.509 | |
| | FDR p | 0.69 | |
| IGF-1 EOR (ng/ml) | N | 141 | 222 |
| | Median (SD) | 136.3 (47.77) | 139.72 (47.87) |
| | p | 0.378 | |
| | FDR p | 0.61 | |
| BDNF EOR (ng/ml) | N | 127 | 204 |
| | Median (SD) | 20.56 (9.02) | 20.95 (8.12) |
| | p | 0.393 | |
| | FDR p | 0.62 | |
| Leptin EOR (ng/ml) | N | 138 | 211 |
| | Median (SD) | 3.18 (2.72) | 3.42 (2.77) |
| | p | 0.317 | |
| | FDR p | 0.53 | |

Shapiro-Wilk test for normality in all dependent variables; Mann-Whitney test or unpaired t-test was implemented; *indicates significances; FDR (false discovery rate) adjusted p following Benjamin-Hochberg (1995) potential false positives marked

*Table S4. Multiple linear regression model baseline biomarkers and baseline pain intensity + BMI + age*

| Model | **Baseline Pain Intensity** | Unstandard. | Standard error | Standard. | t | p | Tol. | VIF |
|---|---|---|---|---|---|---|---|---|
| $M_0$ | (Intercept) | 64.199 | 1.115 | | 57.584 | < .001 | | |
| $M_1$ | (Intercept) | 58.536 | 13.468 | | 4.346 | < .001 | | |
| | AEA baseline | -3.565 | 3.401 | -0.071 | -1.048 | 0.296 | 0.850 | 1.177 |
| | 2AG baseline | -0.558 | 0.926 | -0.040 | -0.603 | 0.547 | 0.853 | 1.172 |
| | IL6 baseline | 0.732 | 0.282 | 0.173 | 2.600 | 0.010* | 0.873 | 1.146 |
| | Cortisol baseline | 0.223 | 0.248 | 0.058 | 0.897 | 0.371 | 0.933 | 1.072 |
| | IGF1 baseline | -0.005 | 0.028 | -0.013 | -0.195 | 0.845 | 0.832 | 1.202 |
| | BDNF baseline | -0.072 | 0.134 | -0.035 | -0.540 | 0.590 | 0.910 | 1.099 |
| | Leptin baseline | 0.289 | 0.447 | 0.048 | 0.648 | 0.518 | 0.698 | 1.432 |
| | BMI baseline | 0.223 | 0.239 | 0.073 | 0.934 | 0.351 | 0.631 | 1.586 |
| | Age | -0.029 | 0.151 | -0.013 | -0.191 | 0.848 | 0.861 | 1.161 |

*Table S5. Multiple linear regression model baseline biomarkers and baseline affective pain + BMI + age*

| Model | **Baseline affective pain** | Unstandard. | Standard error | Standard. | t | p | Tol. | VIF |
|---|---|---|---|---|---|---|---|---|

| | | | | | | | | |
|---|---|---|---|---|---|---|---|---|
| $M_0$ | (Intercept) | 4.179 | 0.224 | | 18.686 | < .001 | | |
| $M_1$ | (Intercept) | 4.770 | 2.698 | | 1.768 | 0.078 | | |
| | AEA baseline | -0.034 | 0.676 | -0.003 | -0.051 | 0.960 | 0.851 | 1.175 |
| | 2AG baseline | -0.360 | 0.184 | -0.133 | -1.960 | 0.051 | 0.857 | 1.167 |
| | IL6 baseline | 0.015 | 0.056 | 0.018 | 0.270 | 0.787 | 0.872 | 1.146 |
| | Cortisol baseline | 0.051 | 0.049 | 0.067 | 1.026 | 0.306 | 0.931 | 1.074 |
| | IGF1 baseline | -0.002 | 0.006 | -0.022 | -0.325 | 0.745 | 0.838 | 1.193 |
| | BDNF baseline | 0.016 | 0.027 | 0.040 | 0.604 | 0.547 | 0.911 | 1.098 |
| | Leptin baseline | 0.146 | 0.089 | 0.122 | 1.639 | 0.103 | 0.706 | 1.417 |
| | BMI baseline | 0.021 | 0.048 | 0.035 | 0.442 | 0.659 | 0.638 | 1.567 |
| | age | -0.043 | 0.030 | -0.096 | -1.420 | 0.157 | 0.863 | 1.159 |

*Table S6. Pearson's Correlations baseline biomarkers*

| Variable | | 2-AG baseline | AEA baseline | IL-6 baseline | Cortisol baseline | IGF-1 baseline | BDNF baseline |
|---|---|---|---|---|---|---|---|
| 1. 2-AG baseline | Pearson's r | — | | | | | |
| | p-value | — | | | | | |
| 2. AEA baseline | Pearson's r | -0.005 | — | | | | |
| | p-value | 0.925 | — | | | | |
| | FDR p | 0.97 | | | | | |
| 3. IL-6 baseline | Pearson's r | 0.172 | 0.227 | — | | | |
| | p-value | < .001* | < .001* | — | | | |
| | FDR p | 0.005 | 0.005 | | | | |
| 4. Cortisol baseline | Pearson's r | 0.065 | 0.035 | 0.061 | — | | |
| | p-value | 0.193 | 0.477 | 0.218 | — | | |
| | FDR p | 0.41 | 0.68 | 0.44 | | | |
| 5. IGF-1 baseline | Pearson's r | -0.139 | -0.059 | -0.174 | -0.058 | — | |
| | p-value | 0.012* | 0.292 | 0.002* | 0.302 | — | |
| | FDR p | 0.03 | 0.52 | 0.009 | 0.52 | | |
| 6. BDNF baseline | Pearson's r | 0.235 | -0.138 | 0.113 | $-5.920\times10^{-4}$ | -0.076 | — |
| | p-value | < .001* | 0.010* | 0.034° | 0.991 | 0.174 | — |
| | FDR p | 0.005 | 0.03 | 0.08° | 0.991 | 0.39 | |
| 7. Leptin baseline | Pearson's r | 0.093 | 0.177 | 0.145 | 0.004 | -0.208 | -0.005 |
| | p-value | 0.085 | 0.001* | 0.007* | 0.937 | < .001* | 0.937 |
| | FDR p | 0.21 | 0.005 | 0.02 | 0.97 | 0.005 | 0.97 |

*indicates significances; FDR adjusted p following Benjamin-Hochberg (1995) potential false positives marked with (°); AEA= Arachidonoylethanolamine, 2-AG=2-arachidonoylglycerol, BMI=Body Mass Index, BDNF=Brain-derived neurotrophic factor, IGF-1= Insulin-like growth factor 1, IL-6= Interleukin-6

*Table S7. Pearson's correlations EOR biomarkers*

| Variable | | 2-AG EOR | AEA EOR | IL-6 EOR | Cortisol EOR | IGF-1 EOR | BDNF EOR |
|---|---|---|---|---|---|---|---|
| 1. 2-AG EOR | Pearson's r | — | | | | | |
| | p-value | — | | | | | |
| 2. AEA EOR | Pearson's r | 0.072 | — | | | | |
| | p-value | 0.189 | — | | | | |
| | FDR p | 0.41 | | | | | |
| 3. IL-6 EOR | Pearson's r | 0.158 | -0.013 | — | | | |
| | p-value | 0.004* | 0.818 | — | | | |
| | FDR p | 0.017 | 0.91 | | | | |
| 4. Cortisol EOR | Pearson's r | 0.024 | 0.002 | 0.010 | — | | |
| | p-value | 0.671 | 0.968 | 0.845 | — | | |
| | FDR p | 0.81 | 0.97 | 0.92 | | | |
| 5. IGF-1 EOR | Pearson's r | -0.086 | -0.026 | -0.126 | 0.138 | — | |
| | p-value | 0.121 | 0.636 | 0.016* | 0.009* | — | |
| | FDR p | 0.29 | 0.78 | 0.04 | 0.03 | | |
| 6. BDNF EOR | Pearson's r | -0.041 | -0.146 | -0.005 | 0.017 | -0.029 | — |
| | p-value | 0.475 | 0.011* | 0.929 | 0.764 | 0.596 | — |
| | FDR p | 0.68 | 0.03 | 0.97 | 0.86 | 0.75 | |
| 7. Leptin EOR | Pearson's r | 0.157 | 0.194 | 0.067 | -0.021 | -0.183 | 0.020 |
| | p-value | 0.005* | < .001* | 0.215 | 0.697 | < .001* | 0.726 |
| | FDR p | 0.02 | 0.005 | 0.44 | 0.83 | 0.005 | 0.84 |

*indicates significances; FDR adjusted p following Benjamin-Hochberg (1995) potential false positives marked with (°); AEA= Arachidonoylethanolamine, 2-AG=2-arachidonoylglycerol, BMI=Body Mass Index, BDNF= Brain-derived neurotrophic factor, EOR=End of rehabilitation, IGF-1= Insulin-like growth factor 1, IL-6= Interleukin-6

*Table S8. Multiple linear regression model significant pre-post changes and GPT*

| Model | **GPT** | Unstandard. | Standard error | Standard. | t | p | Tol. | VIF |
|---|---|---|---|---|---|---|---|---|
| $M_0$ | (Intercept) | 2.695 | 0.099 | | 27.335 | < .001 | | |
| $M_1$ | (Intercept) | 2.963 | 0.131 | | 22.575 | < .001 | | |
| | AEA pre-post | -0.734 | 0.312 | -0.201 | -2.350 | 0.02* | 0.920 | 1.087 |
| | BMI pre-post | $9.375\times10^{-5}$ | $7.416\times10^{-5}$ | 0.104 | 1.264 | 0.208 | 0.983 | 1.018 |
| | Cortisol pre-post | -0.014 | 0.019 | -0.062 | -0.735 | 0.463 | 0.943 | 1.061 |
| | IGF-1 pre-post | 0.003 | 0.003 | 0.088 | 1.061 | 0.291 | 0.979 | 1.021 |
| | Leptin pre-post | -0.162 | 0.071 | -0.193 | -2.278 | 0.024* | 0.938 | 1.066 |

*indicates significances; pre-post: baseline-EOR values; AEA= Arachidonoylethanolamine, 2-AG=2-arachidonoylglycerol, BMI=Body Mass Index, BDNF= Brain-derived neurotrophic factor, EOR=End of rehabilitation, GPT=global performance of treatment, IGF-1= Insulin-like growth factor 1, IL-6= Interleukin-6

*Table S9. Multiple linear regression significant biomarker changes (pre-post) and GPQ Pain intensity change pre-post*

| Model | **Change GPQ Pain Intensity Pre-post** | Unstandard. | Standard error | Standard. | t | p | Tol. | VIF |
|---|---|---|---|---|---|---|---|---|
| $M_0$ | (Intercept) | 11.244 | 1.789 | | 6.285 | < .001 | | |
| $M_1$ | (Intercept) | 8.996 | 2.475 | | 3.634 | < .001 | | |
| | AEA pre-post | 10.544 | 5.969 | 0.153 | 1.766 | 0.080 | 0.926 | 1.080 |
| | BMI pre-post | -0.001 | 0.001 | -0.072 | -0.851 | 0.396 | 0.982 | 1.019 |
| | Cortisol pre-post | -0.720 | 0.366 | -0.169 | -1.969 | 0.051 | 0.950 | 1.052 |
| | IGF-1 pre-post | 0.027 | 0.060 | 0.038 | 0.453 | 0.651 | 0.981 | 1.019 |
| | Leptin pre-post | 1.436 | 1.327 | 0.094 | 1.082 | 0.281 | 0.932 | 1.073 |

*indicates significances; pre-post: baseline-EOR values; AEA= Arachidonoylethanolamine, 2-AG=2-arachidonoylglycerol, BMI=Body Mass Index, BDNF= Brain-derived neurotrophic factor, EOR=End of rehabilitation, GPS=German Pain Society, IGF-1= Insulin-like growth factor 1, IL-6= Interleukin-6

*Table S10. Multiple linear regression significant biomarker changes (pre-post) and GPQ affective pain intensity change pre-post*

| Model | **Change GPQ affective pain Intensity Pre-post** | Unstandard. | Standard error | Standard. | t | p | Tol. | VIF |
|---|---|---|---|---|---|---|---|---|
| $M_0$ | (Intercept) | 1.126 | 0.370 | | 3.040 | 0.003 | | |
| $M_1$ | (Intercept) | 1.352 | 0.510 | | 2.652 | 0.009 | | |
| | AEA pre-post | 1.157 | 1.278 | 0.081 | 0.905 | 0.367 | 0.914 | 1.094 |
| | BMI pre-post | $-5.468\times10^{-4}$ | $2.783\times10^{-4}$ | -0.169 | -1.965 | 0.052 | 0.978 | 1.022 |
| | Cortisol pre-post | -0.082 | 0.075 | -0.095 | -1.085 | 0.280 | 0.955 | 1.047 |
| | IGF-1 pre-post | 0.014 | 0.012 | 0.095 | 1.098 | 0.274 | 0.969 | 1.032 |
| | Leptin pre-post | -0.236 | 0.270 | -0.077 | -0.874 | 0.384 | 0.936 | 1.068 |

*indicates significances, pre-post: baseline-EOR values; AEA= Arachidonoylethanolamine, 2-AG=2-arachidonoylglycerol, BMI=Body Mass Index, BDNF= Brain-derived neurotrophic factor, EOR=End of rehabilitation, GPS=German Pain Society, IGF-1= Insulin-like growth factor 1, IL-6= Interleukin-6

*Table S11. Multiple linear Regression GPT and baseline biomarkers + BMI + EOR pain intensity*

| Model | **GPT** | Unstandard. | Standard error | Standard | t | p | Tol. | VIF |
|---|---|---|---|---|---|---|---|---|
| $M_0$ | (Intercept) | 2.723 | 0.084 | | 32.302 | < .001 | | |
| $M_1$ | (Intercept) | 1.165 | 0.900 | | 1.294 | 0.197 | | |
| | AEA baseline | -0.082 | 0.238 | -0.023 | -0.346 | 0.730 | 0.847 | 1.181 |
| | 2-AG baseline | -0.122 | 0.061 | -0.135 | -1.992 | 0.048* | 0.796 | 1.256 |
| | IL-6 baseline | -0.012 | 0.018 | -0.043 | -0.664 | 0.507 | 0.855 | 1.169 |
| | Cortisol baseline | 0.001 | 0.016 | 0.005 | 0.079 | 0.937 | 0.888 | 1.127 |
| | IGF-1 baseline | 0.001 | 0.002 | 0.043 | 0.668 | 0.505 | 0.865 | 1.156 |
| | BDNF baseline | 0.003 | 0.009 | 0.020 | 0.314 | 0.754 | 0.902 | 1.109 |
| | Leptin baseline | -0.064 | 0.028 | -0.164 | -2.263 | 0.025* | 0.696 | 1.437 |
| | BMI baseline | 0.023 | 0.016 | 0.114 | 1.438 | 0.152 | 0.587 | 1.704 |
| | Age | $-7.786\times10^{-4}$ | 0.010 | -0.005 | -0.081 | 0.936 | 0.861 | 1.161 |

| | | | | | | | | |
|---|---|---|---|---|---|---|---|---|
| | DSF pain intens EOR | 0.025 | 0.003 | 0.493 | 7.839 | < .001* | 0.926 | 1.080 |

Model biomarkers only

| Model | **GPT** | Unstandard. | Standard error | Standard | t | p | Tol. | VIF |
|---|---|---|---|---|---|---|---|---|
| $M_0$ | (Intercept) | 2.731 | 0.084 | | 32.556 | < .001 | | |
| $M_1$ | (Intercept) | 2.839 | 0.574 | | 4.949 | < .001 | | |
| | AEA baseline | -0.101 | 0.251 | -0.028 | -0.403 | 0.687 | 0.948 | 1.055 |
| | 2-AG baseline | -0.207 | 0.068 | -0.225 | -3.065 | 0.002* | 0.864 | 1.157 |
| | IL-6 baseline | 0.017 | 0.020 | 0.062 | 0.854 | 0.394 | 0.894 | 1.119 |
| | Cortisol baseline | 0.007 | 0.017 | 0.027 | 0.397 | 0.692 | 0.983 | 1.017 |
| | IGF-1 baseline | 0.002 | 0.002 | 0.081 | 1.148 | 0.252 | 0.934 | 1.071 |
| | BDNF baseline | $3.872\times10^{-4}$ | 0.010 | 0.003 | 0.037 | 0.970 | 0.910 | 1.099 |
| | Leptin baseline | -0.035 | 0.028 | -0.088 | -1.246 | 0.214 | 0.939 | 1.065 |

*indicates significances; AEA= Arachidonoylethanolamine, 2-AG=2-arachidonoylglycerol, BMI=Body Mass Index, BDNF= Brain-derived neurotrophic factor, EOR=End of rehabilitation, GPT=global performance of treatment, IGF-1= Insulin-like growth factor 1, IL-6= Interleukin-6

*Table S12. Multiple linear Regression GPT and EOR biomarkers + BMI + EOR pain intensity*

| Model | **GPT** | Unstandard. | Standard error | Standard | t | p | Tol. | VIF |
|---|---|---|---|---|---|---|---|---|
| $M_0$ | (Intercept) | 2.706 | 0.100 | | 27.156 | < .001 | | |
| $M_1$ | (Intercept) | 2.662 | 1.112 | | 2.395 | 0.018 | | |
| | AEA EOR | -0.197 | 0.342 | -0.047 | -0.576 | 0.566 | 0.755 | 1.325 |
| | 2-AG EOR | -0.127 | 0.109 | -0.096 | -1.166 | 0.246 | 0.758 | 1.319 |
| | IL-6 EOR | -0.006 | 0.008 | -0.062 | -0.826 | 0.410 | 0.894 | 1.118 |
| | Cortisol EOR | -0.002 | 0.021 | -0.008 | -0.109 | 0.914 | 0.911 | 1.098 |
| | IGF-1 EOR | -0.002 | 0.002 | -0.085 | -1.061 | 0.291 | 0.790 | 1.265 |
| | BDNF EOR | -0.016 | 0.010 | -0.117 | -1.562 | 0.121 | 0.909 | 1.100 |
| | Leptin EOR | -0.057 | 0.036 | -0.136 | -1.581 | 0.116 | 0.688 | 1.453 |
| | BMI EOR | 0.019 | 0.017 | 0.099 | 1.088 | 0.279 | 0.618 | 1.618 |
| | DSF pain intens EOR | 0.025 | 0.004 | 0.529 | 7.145 | < .001* | 0.926 | 1.080 |
| | Age | -0.011 | 0.012 | -0.076 | -0.949 | 0.344 | 0.801 | 1.249 |

Model biomarkers only

| Model | **GPT** | Unstandard. | Standard error | Standard | t | p | Tol. | VIF |
|---|---|---|---|---|---|---|---|---|
| $M_0$ | (Intercept) | 2.710 | 0.086 | | 31.683 | < .001 | | |
| $M_1$ | (Intercept) | 3.098 | 0.575 | | 5.383 | < .001 | | |
| | AEA EOR | 0.178 | 0.322 | 0.040 | 0.553 | 0.581 | 0.949 | 1.054 |
| | 2-AG EOR | -0.209 | 0.100 | -0.158 | -2.090 | 0.038* | 0.873 | 1.146 |
| | IL-6 EOR | $1.169\times10^{-4}$ | 0.009 | $9.526\times10^{-4}$ | 0.013 | 0.990 | 0.924 | 1.082 |
| | Cortisol EOR | 0.009 | 0.019 | 0.032 | 0.444 | 0.658 | 0.969 | 1.032 |
| | IGF-1 EOR | $-8.970\times10^{-5}$ | 0.002 | -0.004 | -0.046 | 0.963 | 0.874 | 1.144 |
| | BDNF EOR | -0.015 | 0.010 | -0.108 | -1.512 | 0.132 | 0.981 | 1.020 |
| | Leptin EOR | -0.011 | 0.033 | -0.026 | -0.343 | 0.732 | 0.872 | 1.147 |

*indicates significances; AEA= Arachidonoylethanolamine, 2-AG=2-arachidonoylglycerol, BMI=Body Mass Index, BDNF= Brain-derived neurotrophic factor, EOR=End of rehabilitation, GPT=global performance of treatment, IGF-1= Insulin-like growth factor 1, IL-6= Interleukin-6

*Table S13. Multiple linear regression EOR GPQ pain intensity and all baseline biomarkers + BMI, Age*

| Model | **EOR GPQ Pain Intensity** | Unstandard. | Standard error | Standard. | t | p | Tol. | VIF |
|---|---|---|---|---|---|---|---|---|
| $M_0$ | (Intercept) | 51.659 | 1.692 | | 30.536 | < .001 | | |
| $M_1$ | (Intercept) | 51.989 | 20.410 | | 2.547 | 0.012 | | |
| | AEA baseline | -7.390 | 5.500 | -0.100 | -1.344 | 0.181 | 0.854 | 1.170 |
| | 2AG baseline | -3.461 | 1.387 | -0.189 | -2.495 | 0.013* | 0.827 | 1.209 |
| | IL6 baseline | 0.872 | 0.403 | 0.159 | 2.167 | 0.031* | 0.881 | 1.135 |
| | Cortisol baseline | 0.523 | 0.365 | 0.104 | 1.433 | 0.153 | 0.897 | 1.115 |
| | IGF-1 baseline | 0.021 | 0.039 | 0.040 | 0.537 | 0.592 | 0.868 | 1.152 |
| | BDNF baseline | -0.142 | 0.216 | -0.048 | -0.659 | 0.511 | 0.898 | 1.114 |
| | Leptin baseline | 0.548 | 0.657 | 0.069 | 0.833 | 0.406 | 0.697 | 1.434 |
| | BMI baseline | -0.039 | 0.364 | -0.010 | -0.108 | 0.914 | 0.589 | 1.696 |
| | age | -0.014 | 0.222 | -0.005 | -0.062 | 0.950 | 0.869 | 1.151 |

Model biomarkers only

| Model | **EOR GPQ Pain Intensity** | Unstandard. | Standard error | Standard. | t | p | Tol. | VIF |
|---|---|---|---|---|---|---|---|---|
| $M_0$ | (Intercept) | 51.571 | 1.678 | | 30.731 | < .001 | | |
| $M_1$ | (Intercept) | 50.153 | 11.520 | | 4.354 | < .001 | | |
| | AEA baseline | -7.888 | 5.164 | -0.107 | -1.527 | 0.128 | 0.945 | 1.058 |
| | 2AG baseline | -3.631 | 1.345 | -0.197 | -2.700 | 0.008* | 0.863 | 1.159 |
| | IL6 baseline | 0.895 | 0.396 | 0.162 | 2.257 | 0.025* | 0.895 | 1.117 |
| | Cortisol baseline | 0.499 | 0.343 | 0.100 | 1.458 | 0.147 | 0.986 | 1.014 |
| | IGF-1 baseline | 0.024 | 0.037 | 0.046 | 0.653 | 0.514 | 0.935 | 1.069 |
| | BDNF baseline | -0.116 | 0.213 | -0.039 | -0.546 | 0.586 | 0.901 | 1.109 |
| | Leptin baseline | 0.557 | 0.560 | 0.070 | 0.993 | 0.322 | 0.934 | 1.070 |

*indicates significances; AEA= Arachidonoylethanolamine, 2-AG=2-arachidonoylglycerol, BMI=Body Mass Index, BDNF= Brain-derived neurotrophic factor, EOR=End of rehabilitation, GPS= German Pain Society, IGF-1= Insulin-like growth factor 1, IL-6= Interleukin-6

*Table S14. Multiple linear Regression GPQ EOR affektive pain and all baseline biomarkers, BMI*

| Model | **GPQ EOR affektive pain** | Unstandard. | Standard error | Standard | t | p | Tol. | VIF |
|---|---|---|---|---|---|---|---|---|
| $M_0$ | (Intercept) | 3.049 | 0.271 | | 11.271 | < .001 | | |
| $M_1$ | (Intercept) | 1.024 | 3.288 | | 0.311 | 0.756 | | |
| | AEA baseline | -1.188 | 0.895 | -0.102 | -1.327 | 0.186 | 0.830 | 1.205 |
| | 2-AG baseline | -0.564 | 0.222 | -0.195 | -2.534 | 0.012* | 0.830 | 1.205 |
| | IL-6 baseline | 0.116 | 0.064 | 0.134 | 1.794 | 0.074 | 0.880 | 1.137 |
| | Cortisol baseline | 0.044 | 0.059 | 0.056 | 0.756 | 0.451 | 0.895 | 1.118 |
| | IGF-1 baseline | 0.006 | 0.006 | 0.077 | 1.020 | 0.309 | 0.864 | 1.157 |
| | BDNF baseline | 0.017 | 0.035 | 0.036 | 0.486 | 0.628 | 0.903 | 1.108 |
| | Leptin baseline | 0.015 | 0.106 | 0.012 | 0.140 | 0.889 | 0.724 | 1.381 |
| | BMI baseline | 0.053 | 0.059 | 0.081 | 0.893 | 0.373 | 0.590 | 1.696 |
| | age | 0.003 | 0.036 | 0.006 | 0.075 | 0.940 | 0.864 | 1.157 |

Model biomarkers only

| Model | **GPQ EOR affektive pain** | Unstandard. | Standard error | Standard | t | p | Tol. | VIF |
|---|---|---|---|---|---|---|---|---|
| $M_0$ | (Intercept) | 3.039 | 0.268 | | 11.334 | < .001 | | |
| $M_1$ | (Intercept) | 2.445 | 1.843 | | 1.326 | 0.186 | | |
| | AEA baseline | -0.951 | 0.829 | -0.082 | -1.147 | 0.253 | 0.944 | 1.059 |
| | 2-AG baseline | -0.548 | 0.216 | -0.189 | -2.543 | 0.012* | 0.866 | 1.155 |
| | IL-6 baseline | 0.127 | 0.063 | 0.146 | 1.996 | 0.047* | 0.895 | 1.117 |
| | Cortisol baseline | 0.028 | 0.055 | 0.036 | 0.516 | 0.607 | 0.987 | 1.013 |
| | IGF-1 baseline | 0.006 | 0.006 | 0.076 | 1.065 | 0.288 | 0.936 | 1.068 |
| | BDNF baseline | 0.020 | 0.034 | 0.043 | 0.586 | 0.559 | 0.907 | 1.103 |
| | Leptin baseline | 0.066 | 0.092 | 0.051 | 0.708 | 0.480 | 0.932 | 1.073 |

*indicates significances; AEA= Arachidonoylethanolamine, 2-AG=2-arachidonoylglycerol, BMI=Body Mass Index, BDNF= Brain-derived neurotrophic factor, EOR=End of rehabilitation, GPS= German Pain Society, IGF-1= Insulin-like growth factor 1, IL-6= Interleukin-6

*Table S15. Multiple linear regression EOR GPS Pain Intensity and all EOR biomarkers + BMI, Age*

| Model | **EOR GPQ Pain Intensity** | Unstandard. | Standard error | Standard. | t | p | Tol. | VIF |
|---|---|---|---|---|---|---|---|---|
| $M_0$ | (Intercept) | 53.242 | 2.120 | | 25.116 | < .001 | | |
| $M_1$ | (Intercept) | 88.774 | 26.914 | | 3.298 | 0.001 | | |
| | AEA EOR | 3.506 | 8.521 | 0.039 | 0.411 | 0.681 | 0.759 | 1.317 |
| | 2-AG EOR | -0.654 | 2.323 | -0.026 | -0.282 | 0.779 | 0.822 | 1.216 |
| | IL-6 EOR | 0.144 | 0.194 | 0.064 | 0.745 | 0.458 | 0.920 | 1.087 |
| | Cortisol EOR | -0.090 | 0.537 | -0.014 | -0.167 | 0.868 | 0.916 | 1.092 |
| | IGF-1 EOR | -0.005 | 0.050 | -0.010 | -0.103 | 0.918 | 0.796 | 1.256 |
| | BDNF EOR | -0.518 | 0.257 | -0.172 | -2.010 | 0.046* | 0.931 | 1.074 |
| | Leptin EOR | 1.448 | 0.881 | 0.160 | 1.644 | 0.102 | 0.718 | 1.393 |
| | BMI EOR | -0.258 | 0.424 | -0.063 | -0.609 | 0.544 | 0.633 | 1.580 |
| | age | -0.438 | 0.296 | -0.136 | -1.480 | 0.141 | 0.809 | 1.236 |

Model biomarkers only

| Model | **EOR GPQ Pain Intensity** | Unstandard. | Standard error | Standard. | t | p | Tol. | VIF |
|---|---|---|---|---|---|---|---|---|
| $M_0$ | (Intercept) | 52.690 | 1.737 | | 30.336 | < .001 | | |
| $M_1$ | (Intercept) | 49.985 | 12.284 | | 4.069 | < .001 | | |
| | AEA EOR | 6.195 | 6.696 | 0.068 | 0.925 | 0.356 | 0.948 | 1.055 |
| | 2-AG EOR | -0.295 | 1.864 | -0.012 | -0.158 | 0.874 | 0.898 | 1.113 |
| | IL-6 EOR | 0.162 | 0.185 | 0.065 | 0.879 | 0.381 | 0.937 | 1.067 |
| | Cortisol EOR | -0.090 | 0.415 | -0.016 | -0.217 | 0.828 | 0.978 | 1.022 |
| | IGF-1 EOR | 0.004 | 0.041 | 0.007 | 0.091 | 0.927 | 0.891 | 1.123 |
| | BDNF EOR | -0.156 | 0.207 | -0.054 | -0.755 | 0.451 | 0.982 | 1.019 |
| | Leptin EOR | 0.667 | 0.670 | 0.075 | 0.996 | 0.320 | 0.880 | 1.136 |

*indicates significances; AEA= Arachidonoylethanolamine, 2-AG=2-arachidonoylglycerol, BMI=Body Mass Index, BDNF= Brain-derived neurotrophic factor, EOR=End of rehabilitation, GPS= German Pain Society, IGF-1= Insulin-like growth factor 1, IL-6= Interleukin-6

*Table S16. Multiple linear regression GPS EOR affektive pain and all EOR biomarkers + BMI, Age*

| Model | **GPQ EOR affektive pain** | Unstandard. | Standard error | Standard. | t | p | Tol. | VIF |
|---|---|---|---|---|---|---|---|---|
| $M_0$ | (Intercept) | 3.291 | 0.342 | | 9.622 | < .001 | | |
| $M_1$ | (Intercept) | 3.740 | 4.282 | | 0.873 | 0.384 | | |
| | AEA EOR | 1.901 | 1.367 | 0.127 | 1.390 | 0.167 | 0.834 | 1.199 |
| | 2-AG EOR | -0.101 | 0.378 | -0.025 | -0.268 | 0.789 | 0.826 | 1.210 |
| | IL-6 EOR | 0.032 | 0.032 | 0.087 | 1.007 | 0.316 | 0.919 | 1.089 |
| | Cortisol EOR | 0.030 | 0.086 | 0.029 | 0.344 | 0.731 | 0.947 | 1.056 |
| | IGF-1 EOR | -0.003 | 0.008 | -0.036 | -0.394 | 0.694 | 0.835 | 1.198 |
| | BDNF EOR | -0.052 | 0.042 | -0.106 | -1.236 | 0.218 | 0.935 | 1.070 |
| | BMI EOR | 0.007 | 0.065 | 0.010 | 0.101 | 0.919 | 0.766 | 1.305 |
| | Age | -0.021 | 0.048 | -0.040 | -0.431 | 0.667 | 0.805 | 1.242 |

Model biomarkers only

| Model | **GPQ EOR affektive pain** | Unstandard. | Standard error | Standard. | t | p | Tol. | VIF |
|---|---|---|---|---|---|---|---|---|
| $M_0$ | (Intercept) | 3.254 | 0.275 | | 11.850 | < .001 | | |
| $M_1$ | (Intercept) | 1.981 | 1.892 | | 1.047 | 0.296 | | |
| | AEA EOR | 1.345 | 1.009 | 0.095 | 1.333 | 0.184 | 0.979 | 1.021 |
| | 2-AG EOR | -0.112 | 0.292 | -0.028 | -0.383 | 0.702 | 0.912 | 1.097 |
| | IL-6 EOR | 0.036 | 0.029 | 0.089 | 1.217 | 0.225 | 0.936 | 1.069 |
| | Cortisol EOR | 0.034 | 0.066 | 0.037 | 0.523 | 0.602 | 0.980 | 1.021 |
| | IGF-1 EOR | $6.315\times10^{-4}$ | 0.006 | 0.007 | 0.100 | 0.920 | 0.931 | 1.074 |
| | BDNF EOR | -0.020 | 0.033 | -0.044 | -0.620 | 0.536 | 0.984 | 1.017 |

*indicates significances; AEA= Arachidonoylethanolamine, 2-AG=2-arachidonoylglycerol, BMI=Body Mass Index, BDNF= Brain-derived neurotrophic factor, EOR=End of rehabilitation, GPS= German Pain Society, IGF-1= Insulin-like growth factor 1, IL-6= Interleukin-6
